# Evaluation of the ischiofemoral space: a case-control study

**DOI:** 10.1590/0100-3984.2018.0095

**Published:** 2019

**Authors:** Antônio Augusto Guimarães Barros, Fernanda Bretz Gomes dos Santos, Carlos César Vassalo, Lincoln Paiva Costa, Sérgio Gonçalves Pereira Couto, Ana Rita da Glória Soares

**Affiliations:** 1 Hospital Madre Teresa, Belo Horizonte, MG, Brazil.

**Keywords:** Hip joint, Ischium/pathology, Joint diseases/diagnostic imaging, Arthralgia/etiology, Magnetic resonance imaging, Articulação do quadril, Ísquio/patologia, Artropatias/diagnóstico por imagem, Artralgia/etiologia, Ressonância magnética

## Abstract

**Objective:**

To determine the size of the ischiofemoral space (IFS) and quadratus femoris space (QFS) in patients with and without ischiofemoral impingement (IFI).

**Materials and Methods:**

Case-control study including consecutive patients submitted to magnetic resonance imaging (MRI) of the hip joint during a three-month period. Patients with deep gluteal pain who tested positive for IFI on at least one clinical test and showed signal changes in the quadratus femoris muscle on MRI were categorized as having a confirmed diagnosis of IFI.

**Results:**

Final sample comprised 50 patients submitted to unilateral MRI of the hip joint. The mean age was 47.3 ± 14.0 years (range, 22-76 years), and 33 (66%) of the patients were women. A diagnosis of IFI was made in 6 patients (12%), all of whom were female. On average, IFS and QFS were significantly smaller in IFI group than in control group (11.1 ± 2.7 mm versus 27.5 ± 6.5 mm and 5.3 ± 1.8 mm versus 18.8 ± 4.8 mm, respectively; *p* < 0.001 for both).

**Conclusion:**

Results of specific clinical tests and MRI findings indicate that the IFS and QFS are significantly reduced in patients with IFI.

## Abstract (pt)

**Objetivo:**

Avaliar, prospectivamente, os valores médios dos espaços isquiofemoral e quadrado femoral em pacientes com impacto isquiofemoral e em um grupo controle.

**Materiais e Métodos:**

Estudo prospectivo incluindo pacientes submetidos a ressonância magnética da articulação do quadril em um intervalo de três meses. Os pacientes com dor glútea profunda, com pelo menos um teste clínico positivo para impacto isquiofemoral e alterações de sinal no músculo quadrado femoral na ressonância magnética, foram diagnosticados com impacto isquiofemoral.

**Resultados:**

A amostra final consistiu de 50 pacientes submetidos a ressonância magnética unilateral da articulação do quadril. A idade média foi 47,3 ± 14,0 anos (intervalo de 22 a 76 anos) e 33 (66%) eram mulheres. O diagnóstico de impacto isquiofemoral foi observado em 6 (12%) pacientes, todas mulheres. Os pacientes com impacto isquiofemoral mostraram redução significativa nos espaços isquiofemoral e quadrado femoral quando comparados ao grupo controle: 11,1 ± 2,7 mm versus 27,5 ± 6,5 mm e 5,3 ± 1,8 mm versus 18,8 ± 4,8 mm, respectivamente (*p* < 0,001 para ambos os grupos).

**Conclusão:**

Os pacientes com diagnóstico de impacto isquiofemoral apresentaram redução significativa dos espaços isquiofemoral e quadrado femoral após análise prospectiva baseada em testes clínicos específicos e ressonância magnética.

## INTRODUCTION

**Figure 1:**
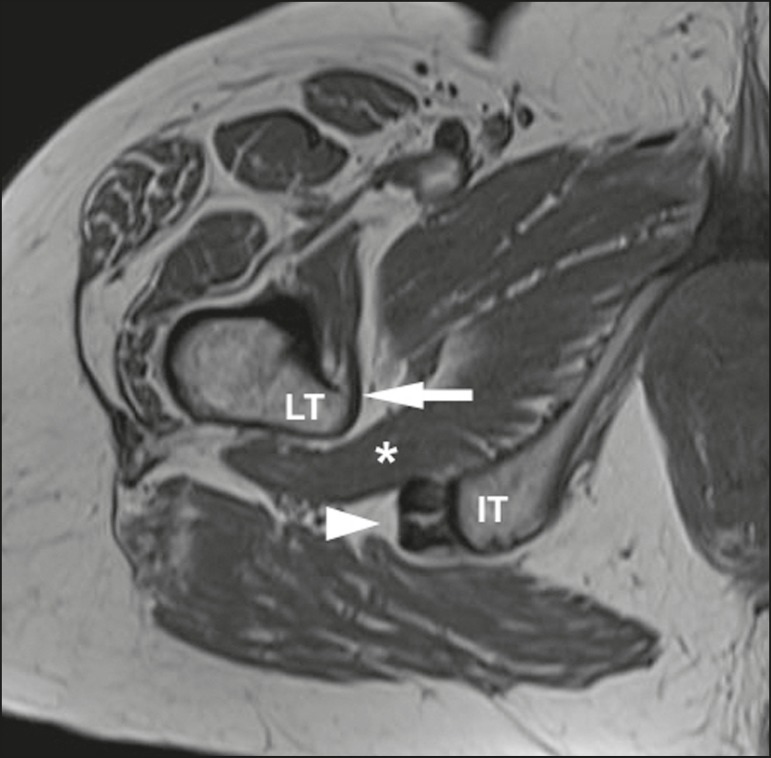
Axial T1-weighted MRI sequence showing the anatomy of the IFS in sections: iliopsoas tendon (arrow), hamstring tendons (arrowhead), and quadratus femoris muscle (asterisk). LT, lesser trochanter; IT, ischial tuberosity.

Ischiofemoral impingement (IFI) was first described in 1977 in three patients with residual pain after total hip arthroplasty^(^^1^^)^. However, this type of impingement has only recently been identified as a potential source of pain in patients with no history of trauma or surgery^(^^2^^)^. IFI is a dynamic condition that leads to compression of the quadratus femoris muscle and pain due to the reduction of the space between the small trochanter and the ischial tuberosity, known as ischiofemoral space (IFS), as depicted in Figure 1. Because of insertion of psoas muscle into lesser trochanter and hamstrings in ischial tuberosity, impingement may also lead to irritation of bursae around those structures^(^^3^^)^. Reduction of IFS has several causes, including coxa valga, coxa profunda, and fracture of the lesser trochanter^(^^3^^)^.

**Figure 2:**
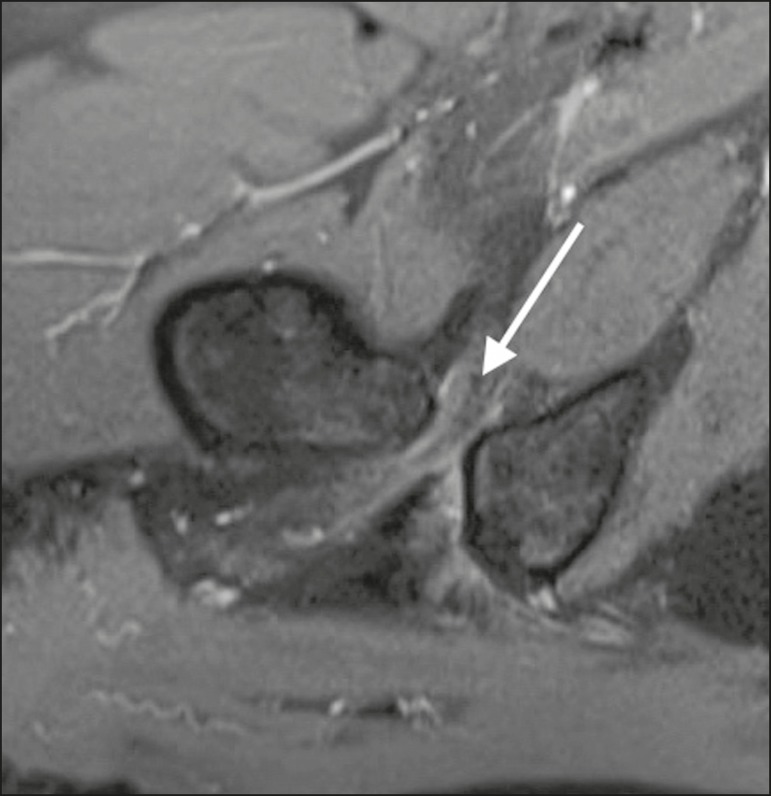
Axial proton density-weighted fat-saturated MRI sequence showing edema of the quadratus femoris muscle and increased quadratus femoris muscle signal intensity, together with smaller IFS and QFS sizes.

Among patients with IFI, the reported mean age is 51-53 years, women predominate, and condition is bilateral in 25-40%^(^^4^^)^. Deep gluteal pain is the main patient complaint, and physical examination is necessary for detection of the impingement. Clinical tests consist of palpation of the IFS, the long-stride walking test, and the IFI test^(^^5^^**,**^^6^^)^. The diagnosis should be confirmed by imaging studies, magnetic resonance imaging (MRI) being the gold standard^(^^7^^)^, which, in patients with IFI, will show a change in the signal of the quadratus femoris muscle, together with reduction of the IFS and the quadratus femoris space (QFS), as shown in Figure 2. However, there are significant differences between individuals with and without IFI in terms of the mean size of the IFS and QFS^(^^8^^)^.

The objective of the present study was to compare asymptomatic patients and patients with symptomatic IFI in terms of the size of the IFS and QFS.

## MATERIALS AND METHODS

This was a case-control study conducted at a hospital with teams specializing in radiology of the musculoskeletal system and in orthopedic hip surgery. We included consecutive patients above 18 years who underwent MRI of hip joint between October and December 2017. Exclusion criteria were as follows: having low back pain; having previously undergone hip surgery; undergoing MRI for the evaluation of traumas (e.g., fractures and muscle injuries), infection, rheumatologic diseases, or hip arthritis; and having metal implants, which can cause image artifacts. Study was approved by local institutional review board, and all participating patients gave written informed consent.

Before undergoing MRI, all patients were examined by a physician trained in clinical orthopedic examination. Symptoms were investigated, and a physical examination was carried out, including clinical tests for the diagnosis of IFI, such as palpation of IFS, long-stride walking test, and IFI test^(^^5^^**,**^^6^^)^. Patients with deep gluteal pain who tested positive for IFI on at least one clinical test and showed signal changes in quadratus femoris muscle in MRI were categorized as having a confirmed diagnosis of IFI. Remaining patients were evaluated as a control group.

**Figure 3:**
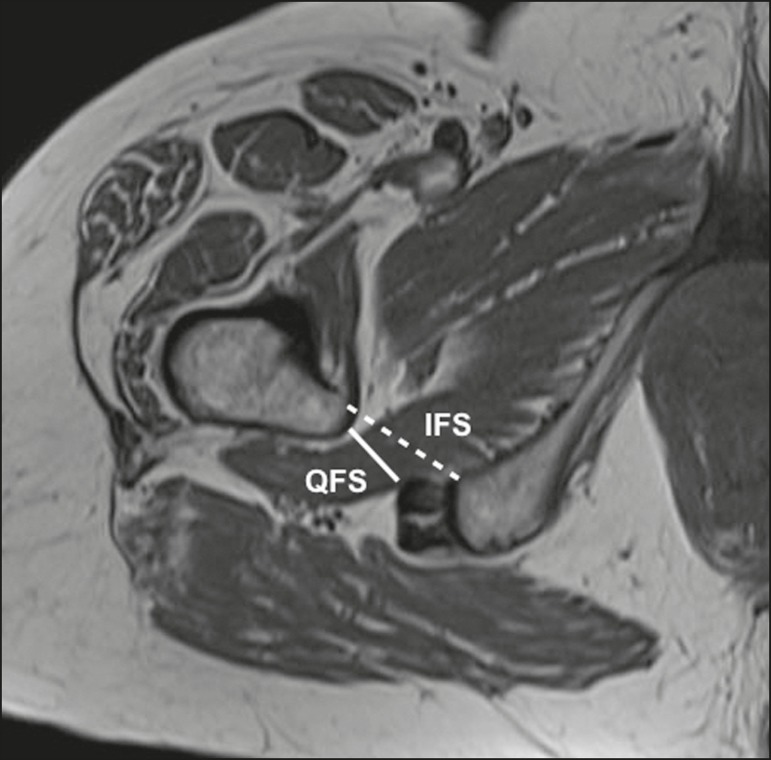
Axial T1-weighted MRI sequence showing the IFS (defined as the shortest distance between the lateral cortex of the ischial tuberosity and the medial cortex of the lesser trochanter) and the QFS (defined as the shortest distance between the hamstring tendons and the medial portion of the lesser trochanter).

To measure IFS and QFS, as well as to identify signal changes in the quadratus femoris muscle, axial MRI examinations were evaluated by two radiologists with experience in imaging of musculoskeletal system. As depicted in Figure 3, IFS is defined as the smallest space between the lateral cortex of the ischial tuberosity and the medial cortex of the lesser trochanter^(^^7^^)^. Figure 3 also shows the QFS, which is defined as the smallest space between the superolateral surface of hamstrings and the posteromedial surface of iliopsoas tendon or lesser trochanter^(^^7^^)^. The radiologists were blinded to results of the clinical evaluations.

All tests were carried out following standardized hip study protocol employed at our facility. Patients were supine, with foot in the neutral position. Following MRI sequences were acquired in a 1.5 T scanner (Avanto; Siemens AG, Berlin, Germany): axial T1-weighted fat-saturated sequences; coronal and axial proton density-weighted, fat-saturated sequences; sagittal and coronal T1-weighted fat-saturated proton density-weighted sequences; proton density-weighted oblique fat-saturated sequences; and axial T1-weighted short-tau inversion-recovery sequences. Slice thickness and field of view were 3.5 mm and 200 mm, respectively.

### Statistical analysis

Statistical analyses were performed with SPSS Statistics software package, version 20.0 (IBM Corp., Armonk, NY, USA). Normal distribution of the continuous variables was determined by the Shapiro-Wilk test. The differences between groups, in the case of continuous variables, were evaluated with the t-test for independent samples and Levene's test for homogeneity of variance or the Mann-Whitney U test, depending on the normality of the data. Fisher's exact test was used in order to evaluate categorical variables. Correlations between continuous variables were evaluated by Spearman's correlation test. For the quantitative variables, the interobserver reproducibility was evaluated by calculating the intraclass correlation coefficient and the corresponding 95% confidence interval (CI).

## RESULTS

**Figure 4:**
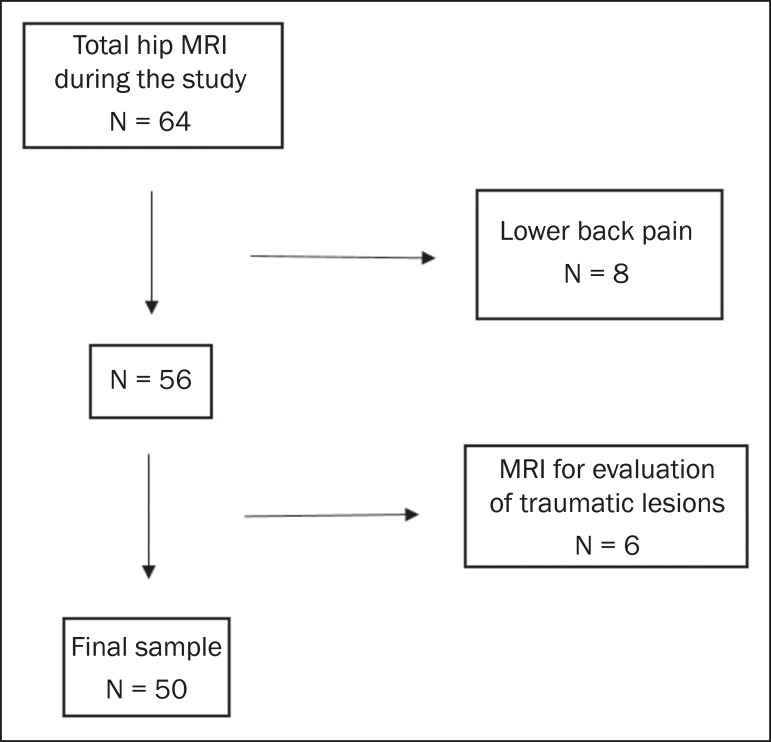
Flowchart of the patient selection process.

After exclusion criteria had been applied, the final sample comprised 50 patients, all of whom were submitted to unilateral MRI of the hip joint (Figure 4). Mean age was 47.3 ± 14.0 years (range, 22-76 years) and 33 (66%) of the patients were women. Mean IFS and QFS measurements were 25.5 ± 8.2 mm and 17.1 ± 6.3 mm, respectively. The intraclass correlation coefficients for the IFS and QFS values were 0.946 (95% CI: 0.907-0.969; *p* < 0.001) and 0.928 (95% CI: 0.877-0.959; *p* < 0.001), respectively. Mean IFS and QFS values were lower among female patients than among male patients (Table 1). A diagnosis of IFI in presence of gluteal pain, positive physical examination, and edema of quadratus femoris muscle was observed in six patients (12%), all of whom were female. Table 2 compares IFS and QFS values between patients with and without IFI.

**Table 1:** Evaluation of the IFS and QFS, by sex.

	Female	Male	
Variable	(n = 33)	(n = 17)	*P*-value
Age (years), mean ± SD	49.4 ± 13.8	43.4 ± 14.0	0.156*
IFS (mm), mean ± SD	23.8 ± 7.9	29.0 ± 8.0	0.032*
QFS (mm), mean ± SD	14.9 ± 5.7	21.6 ± 5.1	< 0.001†

* T-test for independent samples with Levene's test for homogeneity of variance.

† Nonparametric Mann-Whitney U test. SD, standard deviation.

**Table 2:** Comparison of the IFS and QFS in patients with and without IFI.

	IFI group	Control group	
Variable	(n = 6)	(n = 44)	*P*-value
Age (years), mean ± SD	50.5 ± 5.5	46.9 ± 14.8	0.277*
Female, n (%)	6 (100)	27 (61)	0.083†
IFS (mm), mean ± SD	11.1 ± 2.7	27.5 ± 6.5	< 0.001*
QFS (mm), mean ± SD	5.3 ± 1.8	18.8 ± 4.8	< 0.001*

* T-test for independent samples with Levene's test for homogeneity of variance.

† Fisher's exact test. SD, standard deviation.

There was no significant correlation between age and the IFS value (*p* = 0.970) or between age and the QFS value (*p* = 0.294). There was a strongly significant direct correlation between the IFS and QFS values, with a correlation coefficient of 0.767 (*p* < 0.001).

## DISCUSSION

This study corroborates previous findings of literature indicating that reduction of IFS and QFS are associated with symptomatic IFI. However, most of the previous studies evaluating IFS and QFS did not perform specific clinical tests for diagnosis of IFI^(^^4^^**,**^^7^^**,**^^9^^)^.

Torriani et al.^(^^7^^)^ retrospectively compared nine patients with hip pain and abnormal quadratus femoris muscle signal intensity and ten control patients, in terms of the length of those spaces seen on MRI. All patients in that study were women; the mean age was 53 years in the symptomatic group and 67 years in the control group. In the symptomatic group, the location of the symptoms and the findings of the physical examination were not evaluated, and the members of that group might therefore have had hip diseases other than IFI. The IFS was significantly smaller in the symptomatic group than in the control group (13 ± 5 versus 23 ± 8 mm; *p* = 0.002). The QFS was also significantly smaller in the symptomatic group than in the control group (7 ± 3 versus 12 ± 4 mm; *p* = 0.002). Cutoff values of ≤ 17 mm and ≤ 8 mm, respectively, for the IFS and QFS presented the best sensitivity and specificity (83% and 82%, respectively, for both) for identifying hip pain and abnormal quadratus femoris muscle signal intensity on MRI.

In a retrospective analysis, Tosun et al.^(^^4^^)^ compared 70 hips with reported pain and edema of quadratus femoris muscle on MRI and a control group of 38 hips presenting MRI without changes. In symptomatic group, only 31 patients had posterior hip pain and clinical tests for IFI were not performed in any patient. The mean IFS and QFS values, which varied slightly depending on the examiner, were 12.7-13.0 mm and 6.5-6.9 mm, respectively, in the symptomatic patients, and 21.6-21.9 mm and 11.8-13.4 mm, respectively, in the control group patients, the difference between the two groups being statistically significant (*p* < 0.001). 

Ali et al.^(^^9^^)^ described the clinical characteristics of 13 patients (16 hips) with MRI findings suggestive of IFI (abnormal quadratus femoris muscle signal intensity on MRI and narrowing of the IFS). All of the patients in the sample were female, and the mean age was 36 years. When evaluating the symptoms described in the medical records of those patients, the authors found that only six had pain in the deep gluteal region. The seven remaining patients did not present symptoms characteristic of IFI: two presented low back pain and sciatica; and five were asymptomatic in terms of their hips. Therefore, MRI findings alone are not sufficient to diagnose IFI.

As in our study, Gómez-Hoyos et al.^(^^10^^)^ diagnosed patients with IFI based on clinical history, findings on clinical examination, and MRI, although their evaluation was retrospective. During the MRI examinations, the feet were maintained in the functional walking position. The mean size of the IFS on MRI was 11.9 mm and 22.9 mm in the individuals with and without a diagnosis of IFI, respectively (*p* < 0.001). The mean size of the QFS was also lower in patients with a diagnosis of IFI-7.2 mm versus 14.2 mm (*p* < 0.001).

Özdemir et al.^(^^11^^)^ prospectively evaluated the IFS using MRI in 209 asymptomatic volunteers (418 hips). The mean age was 35.9 ± 13.4 years, and 60.3% of the volunteers were men. The mean IFS and QFS values were 25.6 ± 7.5 mm and 15.6 ± 5.4 mm, respectively. The mean values for both spaces were higher in the men than in the women (*p* < 0.01), and there was a negative correlation between IFS length and age (*p* < 0.001). In the present study, the mean values for both spaces were also higher in the men, although age did not correlate significantly with the size of the IFS (*p* = 0.970) or with the size of the QFS (*p* = 0.294). Although the Özdemir et al. study^(^^11^^)^ was prospective and involved a large number of patients, the authors evaluated only asymptomatic volunteers and their sample consisted mostly of men with a mean age of 35.9 years. It is known that IFI mainly affects women around 50 years of age^(^^4^^)^, our study sample therefore being consistent with the epidemiology of this syndrome.

Singer et al.^(^^8^^)^ conducted a meta-analysis involving a collective total of 217 MRI scans of patients with IFI and 140 MRI scans of asymptomatic controls. Mean age was 50.8 ± 12.7 years in the IFI group and 51.6 ± 15 years in the control group. Women accounted for 85.5% of patients in the IFI group and for 70.3% of those in the control group. Mean IFS and QFS values were significantly lower in the IFI group than in the control group-14.9 ± 4.8 mm versus 26.0 ± 7.9 mm and 9.6 ± 3.7 mm versus 15.9 ± 6.0 mm, respectively (*p* < 0.001 for both). Authors found that, for the diagnosis of IFI, an IFS cutoff of ≤ 15 mm had a sensitivity of 76.9%, specificity of 81.0%, and overall accuracy of 78.3%, whereas a QFS cutoff of ≤ 10.0 mm had a sensitivity of 78.7%, specificity of 74.1%, and overall accuracy of 77.1%. They found a strong correlation between a reduction in size of the IFS or QFS and edema/atrophy of the quadratus femoris muscle with ipsilateral hip pain. However, authors concluded that a reduction in size of those spaces alone is insufficient for diagnosis of IFI, stating that gold standard continues to be presence of symptoms and quadratus femoris muscle signal changes on MRI.

Mean IFS values can be influenced by position of hip. Johnson et al.^(^^12^^)^ evaluated the variation in IFS size among three different patient positions in MRI scanner: in supine position with hip in neutral; in supine position with hip flexed; and in prone position. The lowest mean IFS value (21.1 ± 5.6 mm) was observed when patients were in supine position with the hip in neutral. When patients were in prone position, mean IFS value was 28.25 ± 5.91 mm. The highest IFS value (36.9 ± 5.7 mm) was observed when patients were in supine position with hip flexed. Authors found a statistically significant difference between supine position with hip in neutral and prone position, as well as between two supine positions (with the hip in neutral and with the hip flexed). Atkins et al.^(^^13^^)^ evaluated *in vivo* motion of hip and IFS size in 11 asymptomatic participants during weight-bearing activities. Those authors used three-dimensional reconstructions of femur and pelvis generated from MRI, computed tomography, and high-speed dual fluoroscopy. Ten of the 11 participants showed the lowest IFS value during external hip rotation. Mean minimum IFS values found during the activities were 10.8 mm for external rotation, 15.5 mm for level walking, and 15.8 mm for uphill walking, all of which were statistically lower than the mean of 23.7 mm obtained in a static way in the MRI scanner. Mean IFS values found for standing, level walking, and uphill walking were lower in women than in men. These results show that IFS may be smaller during dynamic activities, as well as that values obtained from static images may not represent the minimum size of the space during activities.

The present study has some limitations. Sample was small, and only six patients were included in the IFI group. Patients were not evaluated in relation to height and body weight, variables that could affect the size of the spaces studied. In addition, all patients were evaluated by the same physician, thus precluding the calculation of the reproducibility of the clinical examination. Finally, because it is a dynamic condition, the static evaluation of the spaces studied may not be directly associated with presence of IFI during activities.

## CONCLUSION

This study confirms previous findings showing that patients with IFI diagnosis, considering specific clinical tests, present a significant reduction in the size of the IFS and QFS. We found that mean IFS and QFS values were lower in women than in men.
